# Complement-Independent Modulation of Influenza A Virus Infection by Factor H

**DOI:** 10.3389/fimmu.2020.00355

**Published:** 2020-03-25

**Authors:** Valarmathy Murugaiah, Praveen M. Varghese, Soad M. Saleh, Anthony G. Tsolaki, Salman H. Alrokayan, Haseeb A. Khan, Kate S. Collison, Robert B. Sim, Béatrice Nal, Futwan A. Al-Mohanna, Uday Kishore

**Affiliations:** ^1^Biosciences, College of Health and Life Sciences, Brunel University London, Uxbridge, United Kingdom; ^2^School of Biosciences and Technology, Vellore Institute of Technology, Vellore, India; ^3^Department of Cell Biology, King Faisal Specialist Hospital and Research Centre, Riyadh, Saudi Arabia; ^4^Department of Biochemistry, College of Science, King Saud University, Riyadh, Saudi Arabia; ^5^Department of Biochemistry, University of Oxford, Oxford, United Kingdom

**Keywords:** innate immunity, complement, factor H, vaccinia virus complement control protein, influenza A virus, pseudotyped lentiviral particles, cytokine storm

## Abstract

The complement system is an ancient innate immune defense mechanism that can recognize molecular patterns on the invading pathogens. Factor H, as an inhibitor of the alternative pathway, down-regulates complement activation on the host cell surface. Locally synthesized factor H at the site of infection/injury, including lungs, can act as a pattern recognition molecule without involving complement activation. Here, we report that factor H, a sialic acid binder, interacts with influenza A virus (IAV) and modulates IAV entry, as evident from down-regulation of matrix protein 1 (M1) in H1N1 subtype-infected cells and up-regulation of M1 expression in H3N2-infected A549 cells. Far-western blot revealed that factor H binds hemagglutinin (HA, ~70 kDa), neuraminidase (NA, ~60 kDa), and M1 (~25 kDa). IAV-induced transcriptional levels of IFN-α, TNF-α, IL-12, IL-6, IFN-α, and RANTES were reduced following factor H treatment for the H1N1 subtype at 6 h post-infection. However, for the H3N2 subtype, mRNA levels of these pro-inflammatory cytokines were enhanced. A recombinant form of vaccinia virus complement control protein (VCP), which like factor H, contains CCP modules and has complement-regulatory activity, mirrored the results obtained with factor H. Both factor H (25%), and VCP (45%) were found to reduce luciferase reporter activity in MDCK cells transduced with H1N1 pseudotyped lentiviral particles. Factor H (50%) and VCP (30%) enhanced the luciferase reporter activity for H3N2, suggesting an entry inhibitory role of factor H and VCP against H1N1, but not H3N2. Thus, factor H can modulate IAV infection and inflammatory responses, independent of its complement-related functions.

## Introduction

Influenza A virus (IAV) is a severe respiratory pathogens, belonging to the Orthomyxoviridae family and responsible for outbreaks with high morbidity, and mortality in both humans and animals (1). IAV exhibits antigenic variations within viral surface glycoproteins, including hemagglutinin (HA), and neuraminidase (NA) (2). HA is the most important viral glycoprotein that can bind to sialic acid on host cells, enabling cellular fusion and viral entry (3). HA epitopes are also known to trigger synthesis of neutralizing antibodies by B cells, allowing IAV to escape from immune surveillance, leading to seasonal epidemics (4). NA cleaves sialic acid moieties, which enables release of virions, and promotes IAV dispersion (5). There are around 16 antigenic variants of HA and 9 of NA, which are found in numerous combinations and subtypes (2, 6). Among all subtypes of IAV, H1N1 and H3N2 are the predominant IAV subtypes for seasonal flu in humans, with annual epidemics estimated to result in ~5 million cases of severe illness and half a million respiratory deaths (7). Current circulating human IAV strains are likely to continue acquiring mutations resulting in antigenic drift and shift of HA and NA glycoproteins (8, 9), which enhance the virus' ability to evade host immune system, promoting rapid viral invasion and replication.

Airway and alveolar epithelial cells are the primary targets for IAV, which uses sialic acid as receptors, causing damage to the alveolar epithelium (10, 11). Individuals infected with IAV may become susceptible to acute respiratory distress syndrome (ARDS) (12, 13). Lung epithelial cells express mucin glycoproteins, such as MUC5AC, MUC5B, and MUC1, which play an important role in restricting IAV infectivity (14–16). These mucins are rich in sialic acid, serve as viral receptors, and restrict viral binding to the target cells (16–18). However, NA can attenuate the biological activity of these mucins (18, 19). The D151G mutation is a well-known mutation in NA glycoprotein, which is responsible for its interaction with human α2-6 and avian α2-3 linked sialic acid, mediating the H3N2 viral association with sialic acid receptors. However, this mutation is reported to reduce the enzymatic action of NA that is required for HA detachment from its receptors (19, 20). Early defense against invading IAV by the innate immune system is crucial in limiting viral replication and invasion. The complement system, a major humoral wing of innate immunity, offers a crucial protective mechanism against IAV infection (21). This includes neutralization, aggregation, opsonization and lysis of viral particles, and induction of phagocytosis via complement receptors (22, 23). A number of *in vitro* and *in vivo* studies have established the protective role of the complement system against IAV (24–27).

In the alternative pathway of the complement system, factor H is an important negative regulator that interacts with negatively charged surfaces containing sialic acids and glycosaminoglycans, and protects cellular structures from C3 convertase formation, hence diminishing complement activation. Factor H is a soluble, 155 kDa glycoprotein present at a concentration of 128–654 μg/ml in human plasma (28). It is composed of 20 complement control protein (CCP) modules with different ligand binding properties. There is plenty of evidence of the local extrahepatic synthesis of factor H [reviewed in (30)]. Factor H binds to many pathogens via charge interactions (29), and for pathogens, surface-bound factor H may be of benefit for their survival. Factor H binds to sialic acids on *Neisseria gonorrhoeae* (30) and the outer surface of OspE of *Borrelia burgdorferi*, providing complement resistance to these pathogenic microbes. Its interaction with soluble West Nile virus NS1 protein has also been described (31). Furthermore, *Plasmodium falciparum* binds factor H and factor H-like protein 1 (FHL-1) to prevent complement-mediated lysis in the mosquito midgut via the plasmodial transmembrane gliding-associated protein 50 (GAP50) (32). Factor H can also bind to the surface of mycobacteria, restrict their uptake by macrophages, and modulate pro-inflammatory cytokine responses (33).

Viruses employ diverse strategies to protect their viral lipid envelopes from complement lysis by encoding or recruiting complement inhibitors, with structural and functional similarities to complement control proteins (CCP). Vaccinia virus complement control protein (VCP) is a well-known complement inhibitor, secreted by vaccinia virus infected cells. VCP has inhibitory activity for both classical and alternative pathways (34). Further examples of viral regulation of complement includes binding of West Nile virus non-structural protein (NS1) to factor H, or association of Nipah virions with factor I, thus restricting complement activation (31, 35). In addition, NS1 serves as a key inhibitor of innate immunity as it blocks the synthesis and signaling of type 1 interferons (IFNs) (36). NS1 also induces apoptosis in human airway epithelial cells during IAV infection via caspase-dependent mechanisms (37).

Since factor H can bind to sialic acids, a natural ligand for IAV, it is of interest to examine potential influence of factor H in competitively inhibiting IAV interaction with host cell surfaces. This study was designed to investigate the complement independent functions of factor H in the regulation of IAV infection *in vitro*. Here, we report the ability of human factor H and recombinant VCP to act as entry inhibitors of IAV.

## Materials and Methods

### Viruses and Reagents

The IAV subtypes used in this study, including the pH1N1 (A/England/2009) and H3N2 (A/HK/1999), were provided by Wendy Barclay (Imperial College, London) and Leo Poon (University of Hong Kong), respectively. The plasmids used for the production of H1N1 and H3N2 pseudo-typed viral particles were obtained from Addgene; pHIV-Luciferase (Addgene plasmid #21375); psPAX2 (Addgene plasmid # 12260); and Vesicular Stomatitis Virus (VSV-G) (Addgene plasmid #8454). pcDNA3.1-swineH1-flag (H1 from swine H1N1 A/California/04/09) (Invitrogen), pcDNA3.1-swine N1-flag (N1 from swine H1N1 A/California/04/09), and pCDNA H3 (from A/Denmark/70/03 (H3N2) (Invitrogen) were obtained commercially. pI.18-N2 [N2 from A/Texas/50/2012/(H3N2)] plasmid was a gift from Nigel Temperton (University of Kent). Anti-influenza antibodies used were obtained from BEI Resources, NIAID, NIH, USA, and used as previously described (38); these include polyclonal anti-influenza Virus H3 HA, A/Hong Kong/1/1968 and monoclonal anti-influenza virus H1 HA, A/California /04/2009.

### Purification of Human Complement Factor H

Complement factor H was purified from human plasma, as described previously (39), using an affinity column made up of a monoclonal antibody against human factor H (MRCOX23) coupled to CNBr-activated Sepharose (GE healthcare, UK). Freshly thawed human plasma (Fisher Scientific) was adjusted to 5 mM EDTA, pH 8, and dialyzed overnight against Buffer I (25 mM Tris-HCL, 140 mM NaCl, and 0.5 mM EDTA, pH 7.5). MRCOX23 Sepharose column was washed with three bed volumes of buffer I, and dialyzed plasma was passed through the column. The column was then washed again with the same buffer and factor H was eluted using 3 M MgCl_2_, pH 6.8. The eluted fractions were dialyzed against H_2_O overnight, followed by 10 mM potassium phosphate pH 7.4. The samples were then analyzed for purity by 12% v/v SDS-PAGE.

### Purification of Recombinant Vaccinia Virus Complement Control Protein Expressed in HEK293 Cell Line

The VCP gene (accession X13166.1) was codon-optimized for expression in human embryonic kidney (HEK) cells by GeneArt® using GeneOptimizer® (Geneart GmbH, Regensburg). For lentiviral expression, amplified VCP cDNA was ligated into the pLenti6/V5-D-TOPO vector, using the ViraPower Lentiviral Directional TOPO Expression kit, according to the manufacturer's instructions (K4950-00 Invitrogen Corp, Carlsbad CA). Following transformation into DH5α chemically competent *E. coli* cells, a number of colonies were analyzed for correct insertion and orientation using colony PCR. Transient transfection of the plasmid pLenti6/V5/VCP in HeLa cells and indirect immunofluorescence using anti-V5 antibody (Invitrogen # R960-25) was performed to verify the VCP expression. Replication-incompetent lentiviral stock was made by co-transfection with the ViraPower^TM^ Packaging Mix (pLP1, pLP2, and pLP/VSVG: K4975-00, Invitrogen Corp) in HEK 293FT cells (ATCC CRL-1573) cells using Lipofectamine 2000® reagent (Life Technologies Inc.), according to the manufacturer's instructions. Forty-eight hours after co-transfection, viral supernatant was collected, concentrated by centrifugation, and the titer was determined using standard procedures. A number of stable HEK 293FT cell lines expressing VCP were generated under neomycin selection (0.5 g/L) and screened for VCP expression by Western blot analysis. Three clones of HEK-293 cells secreting high levels of the VCP were selected and cultured. Secreted VCP was purified using a heparin column. Column-bound proteins were eluted with a linear salt gradient (0–0.5 M NaCl). Fractions were collected and analyzed via SDS-PAGE and western blotting.

### Cell Culture and Treatments

Madin Darby Canine Kidney (MDCK) and Adenocarcinomic human alveolar basal epithelial cells (A549) cells (ATCC, Rockville, MD, USA) were cultured in complete DMEM media, supplemented with 10% v/v fetal calf serum, 2 mM L-glutamine, and penicillin (100 U/ml)/streptomycin (100 μg/ml) (Thermo Fisher), at 37°C under 5% v/v CO_2_, with fresh complete medium added every 2–3 days until 80% confluence was reached. Cell lines used in this study were subjected to a maximum of 7 passages for *in vitro* experiments.

### Production of IAV Subtypes and Pseudotyped Viral Particles, and TCID50 (Median Tissue Culture Infectious Dose) Assay

50,000 MDCK cells at 80% confluency were infected either with pH1N1 (2 × 10^4^) or H3N2 (3.3 × 10^4^) particles and incubated in complete DMEM medium at 37°C for 1 h. Unbound viral particles were removed and replaced with infection medium, composed of DMEM with 0.3% bovine serum albumin (BSA), 1% penicillin/streptomycin, and 1 μg/ml of l-1-tosylamido-2-phenylethyl chloromethyl ketone (TPCK)— Trypsin (Sigma –Aldrich), and incubated for 3 days under culture conditions, as mentioned above. Post infection, supernatant was subjected to ultra-centrifugation (25,000 × g) for 90 min at 4°C. Purified viral particles were then re-suspended in PBS, and purity of the virus was analyzed by SDS-PAGE and western blotting. Production of pseudotyped particles was carried out, as described earlier (38). Briefly, HEK293T cells were co-transfected with 20 μg of respective IAV pCDNAs, including pcDNA3.1-swineH1-flag (H1 from swine H1N1 A/California/04/09) (Invitrogen), pcDNA3.1-swine N1-flag (N1 from swine H1N1 A/California/04/09) (Invitrogen), pcDNA-H3 [H3 from A/Denmark/70/03/(H3N2)], pI.18-N2 [N2 from A/Texas/50/2012/(H3N2)], pHIV-Luciferase backbone (Addgene), and psPAX2 (Addgene). VSV-G was generated similarly, without H1N1 and H3N2 pcDNAs. The released H1N1, H3N2, and VSV-G pseudotyped lentiviral particles were harvested in the supernatant at 48 h. Harvested supernatant was centrifuged at 5,000 × g for 20 min, and the clear supernatant without any debris was concentrated using ultra centrifugation (25,000 × g) for 90 min. The ultra-centrifuged lentiviral particles were re-suspended in sterile PBS and analyzed via TCID50, western blotting and luciferase activity assay. TCID50 assay was carried out to determine the viral titer and cytopathic effects of infected cells (40). Briefly, MDCK cells (1 × 10^5^) were infected with either purified pH1N1 and H3N2 viral parties or pseudotyped lentiviral particles, incubated at 37°C for 3 days under 5% v/v CO_2_, until a cytopathic effect was observed in terms of structural changes in MDCK cells caused by viral invasion.

### Hemagglutination Inhibition Assay

In a 96 microtiter well plate, 25 μl of PBS was added to each well. In the first column, a starting concentration (total volume 50 μl) of 20 μg of factor H was serially diluted (25 μl) to achieve final quantities of 20, 10, 5, and 2.5 μg of factor H per well. Twenty-five microliters of respective IAV subtype particles were added to wells, except for PBS control wells, at the dilutions corresponding to their respective HA titer to initiate hemagglutination. Plates were gently mixed and incubated at 37°C for 1 h. Fifty microliter of 0.75% v/v guinea pig RBCs was added to each well; plates were gently mixed and incubated at room temperature for 1 h. Inhibition of hemagglutination appeared as a halo or circle of settled cells in the center of round-bottomed wells. Absence of inhibition was observed as a uniform red color across the well (hemagglutination).

### ELISA to Detect Interaction of Factor H and VCP With IAV Subtypes

Factor H or VCP (5, 2.5, 1.25, and 0.625 μg/well in 100 μl volume) were coated onto 96-well microtiter plates using carbonate-bicarbonate buffer (CBC), pH 9.6, and incubated at 4°C overnight. After washing the microtiter wells with PBS, the wells were blocked with 2% w/v BSA in PBS and incubated at 37°C for 2 h, followed by three PBST (PBS + 0.05% v/v Tween 20) washes. Twenty microliters of H1N1 or H3N2 virus (1.36 × 10^6^ pfu/ml) in PBS were added to each well and incubated at 37°C for 2 h in the presence of 5 mM CaCl_2_. VSV-G pseudotyped lentivirus was used as a negative control. Following PBST washes, the corresponding wells were incubated with primary antibodies (100 μl/well): polyclonal anti-influenza virus H3 and monoclonal anti-influenza virus H1 (1:5,000) (BEI-Resources). The wells were again washed with PBST three times and probed with Protein A-HRP-conjugate, or goat anti-mouse IgG-horseradish peroxidase (HRP)-conjugate (1:5,000) (Fisher Scientific), followed by incubation at 37°C for 1 h. Color was developed using 3,3′, 5,5′-tetramethylbenzidine (TMB) substrate (Sigma-Aldrich), and reaction was stopped using 1M H_2_SO_4_, followed by measuring absorbance at 450 nm, using iMark™ microplate absorbance reader (Bio-Rad).

### Cell Binding Assay to Detect IAV Interference With Factor H and VCP

A549 cells (1 × 10^5^ cells/well) were seeded in 96 microtiter wells and incubated at 37°C overnight in 5% v/v CO_2_. Once 80% confluency was reached, the cells were washed twice with sterile PBS. Factor H or VCP (10, 5, 2.5, 1.25 μg/ml), pre-incubated with H1N1 and H3N2 (1.36 × 10^6^ pfu/ml) IAV subtypes, were added to the wells (in 5 mM CaCl_2_) and incubated at room temperature for 2 h. BSA was used as a negative control. Following washes with PBS three times, the corresponding wells were fixed with 4% v/v paraformaldehyde (Fisher Scientific) for 5 min at room temperature. The wells were then blocked with 2% w/v BSA for 2 h at 37°C. Polyclonal anti-influenza virus H3 (BEI-Resources) and monoclonal anti-influenza H1 (BEI-Resources) were added to the appropriate wells and incubated at 37°C for 1 h. After gentle washes with PBSST, the wells were probed with protein A-HRP conjugate or goat anti-mouse IgG-HRP-conjugate (Thermo-Fisher) diluted in PBS in 1:5,000 dilution and incubated at 37°C for 1 h. The wells were washed again with PBST; the color was developed by adding TMB substrate and the reaction was stopped by using 1M H_2_SO_4_. The absorbance was read at 450 nm using an ELISA plate reader.

### Far-Western Blotting

Purified H1N1/H3N2 (1.36 × 10^6^ pfu/ml) virus particles were run on a 12% (w/v) SDS-PAGE, and transferred onto a PVDF membrane for 2 h at 320 mA in transfer buffer (25 mM Tris–HCl, pH 7.5, 20% v/v methanol, and 190 mM glycine). Membrane was blocked with PBS+5% w/v BSA (Sigma-Aldrich) at room temperature, followed by PBST washes. The membrane was then incubated with 10 μg/ml of factor H or VCP overnight at 4°C, and probed appropriately with either monoclonal mouse anti-human factor H (MRCOX23) (MRC Immunochemistry Unit, Oxford) (1 mg/ml) or rabbit anti-VCP polyclonal antibody (0.5 mg/ml) (King Faisal Specialist Hospital and Research Center, Saudi Arabia) at room temperature for 1 h. Following PBST washes (three times, 10 min each), the membrane was incubated with secondary antibody, rabbit anti-mouse IgG HRP conjugate (1:1,000) (Sigma-Aldrich) or Protein A-HRP-conjugate for 1 h at room temperature. The secondary antibody was removed, followed by PBST washes; then the membrane was developed using 3,3′-diaminobenzidine (DAB).

### Infection Assay for Extracting RNA

A549 (5 × 10^5^/well) cells were cultured in complete DMEM and grown overnight at 37°C in a CO_2_ incubator. Once 85% cell confluency was reached, the cells were washed gently with fresh PBS and replaced with DMEM without FBS. Forty μg/ml of factor H or VCP was added to corresponding wells, with a MOI = 1:1 of pH1N1, and H3N2 or pseudotyped viral particles (333 μl/ml) at room temperature for 1 h and at 4°C for another hour. The unbound virus and protein were removed by pipetting out the supernatant. The cells were washed gently again with PBS, and placed in 1 ml of infection medium to initiate viral infection of the host cells being incubated for 2 and 6 h. After removing the supernatant, the infected cells were washed with PBS and detached using 2 × Trypsin-EDTA (0.5%) (Fisher Scientific), centrifuged at 1,500 x g for 5 min, and the cell pellet was frozen at −80°C for RNA extraction.

### Quantitative RT-PCR Analysis

The virus infected cell pellets were lysed using lysis buffer (50 mM Tris–HCl, pH 7.5, 200 mM NaCl, 5 mM EDTA pH 8, 0.1% v/v Triton X-100). GenElute Mammalian Total RNA Purification Kit (Sigma-Aldrich) was used to extract the total RNA, as per the manufacturer's instructions. Once RNA was extracted, DNase I (Sigma-Aldrich) treatment was performed to remove any DNA contaminants, followed by quantifying the amount of RNA at A260 nm using a NanoDrop 2000/2000c (Fisher-Scientific). The purity of RNA was assessed using the ratio A260/A280. Two micrograms of total RNA was used to synthesize cDNA, using High Capacity RNA to cDNA Kit (Applied Biosystems) and cDNA conversion was performed. The primer BLAST software (Basic Local Alignment Search Tool) was used to design primer sequences as listed in Table 1. The qRT-PCR assay was performed using the StepOne Plus system (Applied Biosciences). Each qPCR reaction was conducted in triplicates, containing 75 nM of forward and reverse primers, 5 μl Power SYBR Green MasterMix (Applied Biosystems), and 500 ng of cDNA. qPCR samples were run for 50°C and 95°C for 2 and 10 min, followed by running the amplification template for 40 cycles, each cycle involving 15 s at 95°C and 1 min at 60°C. Eighteen seconds RNA was used as an endogenous control to normalize the gene expression.

**Table 1:** Primers used for target genes in qPCR.

**Target**	**Forward primer**	**Reverse primer**
18S rRNA	5′-ATGGCCGTTCTTAGTTGGTG-3′	5′-CGCTGAGCCAGTCAGTGTAG-3′
IL-6	5′-GAAAGCAGCAAAGAGGCACT-3′	5′-TTTCACCAGGCAAGTCTCCT-3′
IL-12	5′-AACTTGCAGCTGAAGCCATT-3′	5′-GACCTGAACGCAGAATGTCA-3′
TNF-α	5′-AGCCCATGTTGTAGCAAACC-3′	5′-TGAGGTACAGGCCCTCTGAT-3′
M1	5′AAACATATGTCTGATAACGAAGGAGAACAGTTCTT-3′	5′GCTGAATTCTACCTCATGGTCTTCTTGA-3′
RANTES	5'-GCGGGTACCATGAAGATCTCTG-3'	5'-GGGTCAGAATCAAGAAACCCTC-3'
IFN-α	5′-TTT CTC CTG CCT GAA GGA CAG-3′	5′-GCT CAT GAT TTC TGC TCT GAC A-3′

### Statistical Analysis

The graphs were generated using the GraphPad Prism 6.0 software, A one-way ANOVA test was carried out for statistical significance. Significant values were considered based on ^*^p < 0.05, ^**^p < 0.01, and ^***^p < 0.001, between treated and untreated conditions. Error bars show the SD or SEM, as indicated in the figure legends.

## Results

### Factor H Inhibits Hemagglutination of Guinea Pig Red Blood Cells by IAV

**Figure 1:**
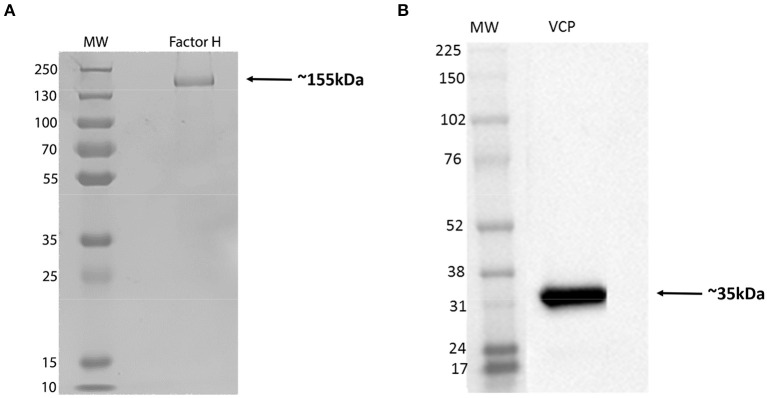
12% (w/v) SDS-PAGE analysis of complement factor H purified from human plasma **(A)**, and a recombinant form of vaccinia virus complement control protein (VCP) expressed in HEK 293FT cells **(B)**. The purified protein was run under reducing conditions: Factor H appears as ~155 kDa band, and VCP was observed at ~35 kDa.

**Figure 2:**
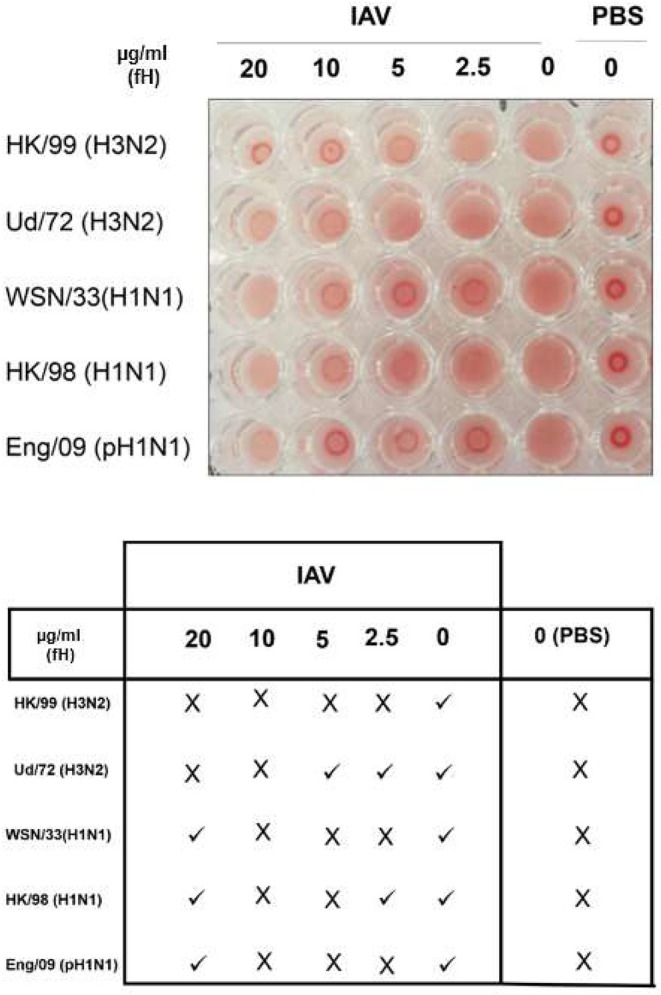
Hemagglutination assay. Inhibition of hemagglutination by purified human factor H was tested on human IAV subtypes. Guinea pig red blood cells were incubated with either PBS or IAV subtypes with and without various concentrations of factor H (20, 10, 5, and 2.5 μg).

Factor H was purified from human plasma, using monoclonal MRCOX23 Sepharose affinity column (39). The purity of the eluted protein was confirmed by SDS-PAGE; the molecular weight of factor H was evident at ~155 kDa (Figure 1A). Recombinant VCP appeared pure and migrated at ~35 kDa (Figure 1B). Guinea pig RBCs were used to determine if addition of factor H would inhibit hemagglutination by IAV subtypes (Figure 2). Five IAV strains of H3N2 or H1N1 subtypes were tested. The basic principle of this assay relies on the specific feature of envelope viral glycoproteins that can interact with sialic acid on RBCs. In the absence of viral particles, RBCs sedimented under gravity, forming a distinct red halo at the bottom of the microtiter well (Figure 2). However, in the presence of viral particles, glycoproteins of virus (HA or NA in this case) interact and bind with sialic acid on RBCs, forming clumps, leading to a lattice formation, where RBCs remain in suspension. Factor H partially inhibited the hemagglutination of IAV strains. Low concentration of factor H (2.5 μg/ml) was more potent in inhibiting hemagglutination of H1N1 than H3N2 strains. Hemagglutination of all strains was effective using 10 μg/ml of factor H. It is possible that factor H binds to the IAV particles, restricting the binding of virus to RBCs, allowing them to form a halo at the bottom of the wells. PBS alone, in the absence of IAV particles, led to the formation of a halo at the bottom of the microtiter well, suggesting no hemagglutination.

### Factor H and VCP Bind IAV Envelope Glycoproteins

**Figure 3:**
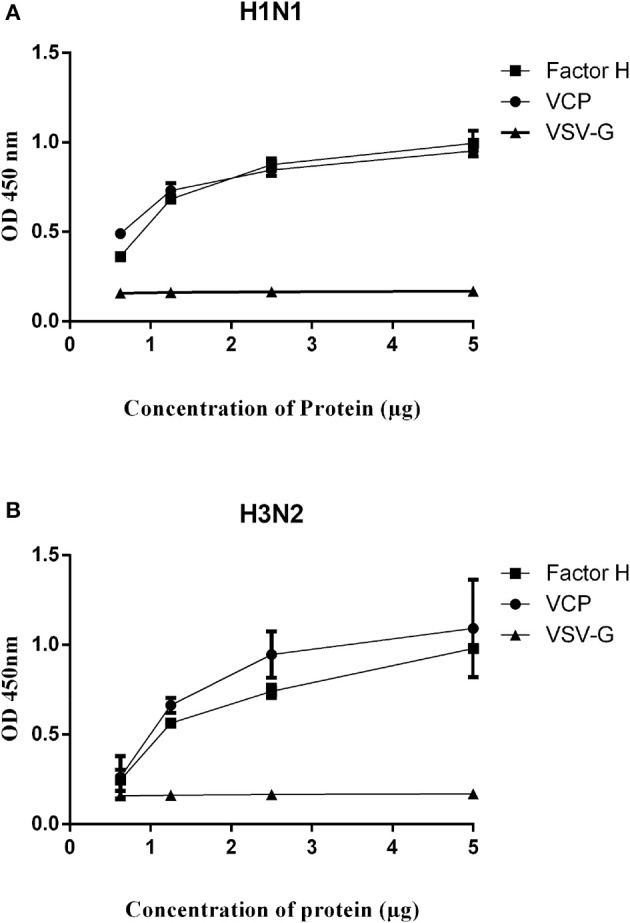
Binding between factor H or VCP with H1N1 **(A)**, and H3N2 **(B)** IAV subtypes were determined via ELISA. Microtiter wells were coated with various concentrations of factor H, VCP or VSV-G (0.625, 1.25, 2.5, and 5 μg/well) in carbonate bicarbonate buffer, pH 9.6 overnight at 4°C. H1N1 or H3N2 virus (1.36 × 10^6^ pfu/ml) was added to each well in the presence of 5 mM CaCl_2_ and probed with either monoclonal anti-influenza virus H1 or polyclonal anti-influenza virus H3 antibody. VSV-G pseudotyped particles were used as a negative RNA virus control. The data were expressed as mean of three independent experiments done in triplicates ± SEM.

**Figure 4:**
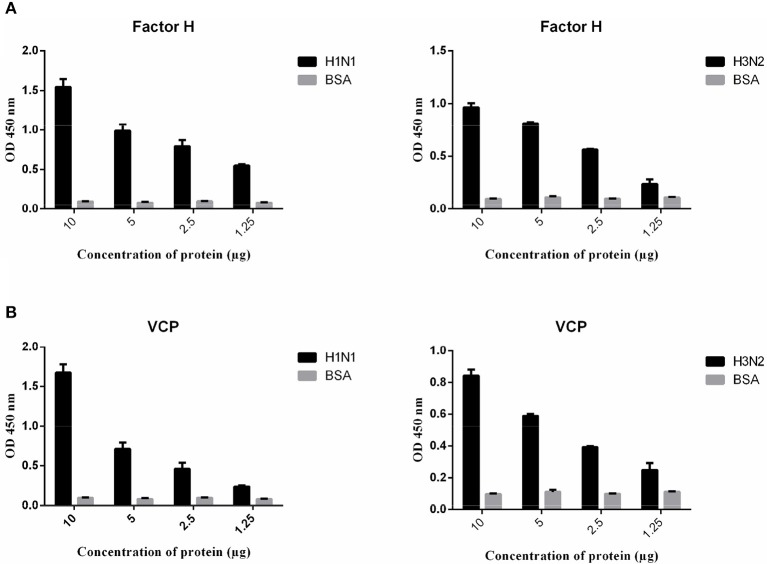
Cell-binding assay to show binding of factor H **(A)** and VCP **(B)** to A549 cells challenged with H1N1 and H3N2. A549 cells (1 × 10^5^ cells/ml) were seeded in a 96-well microtiter plate and incubated at 37°C overnight. Decreasing concentrations of factor H and VCP (10, 5, 2.5, and 1.25 μg), pre-incubated with IAV subtypes, were added to the corresponding wells, and incubated at room temperature for 1 h. After removing unbound protein and viral particles, the wells were fixed with 4% v/v paraformaldehyde, and probed with monoclonal anti-influenza virus H1 or polyclonal anti-influenza virus H3 antibodies. BSA was used as a negative control protein. Three independent experiments were carried out in triplicates and error bars expressed as ± SEM.

**Figure 5:**
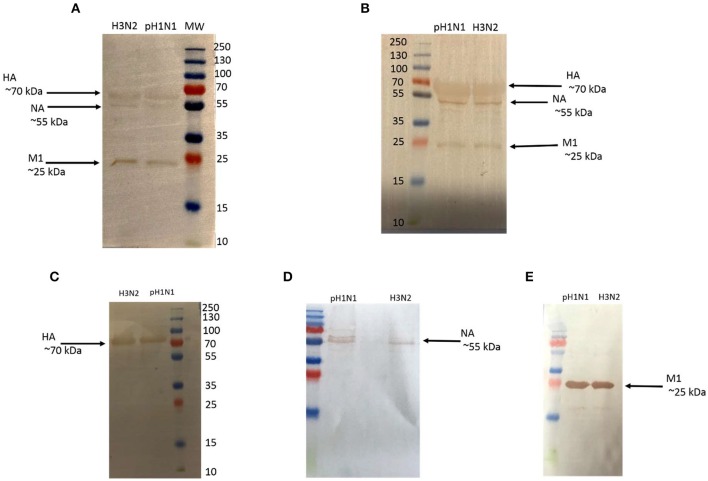
Far western blot analysis to show factor H **(A)** and VCP **(B)** binding with purified H1N1 and H3N2. IAV subtypes (1.36 × 10^6^pfu/ ml) were first run on the SDS-PAGE under reducing conditions and transferred onto a nitrocellulose membrane. The transferred membrane was then incubated with 10 μg/ml of factor H or VCP. The membrane was probed with either monoclonal mouse anti-human factor H or polyclonal rabbit anti-VCP antibody. Factor H and VCP bound to HA (~70 kDa), NA (~55 kDa), and M1 (~25 kDa) in the case of both IAV subtypes. The identities of factor H and VCP bound IAV glycoproteins were validated using a separate blot that was directly probed with monoclonal anti-HA **(C)**, anti-NA **(D)**, and anti-M1 **(E)** antibodies.

Since factor H was able to inhibit hemagglutination by H1N1 and H3N2 IAV subtypes, a direct ELISA was set up with factor H -coated plates to establish possible interaction with IAV viruses (Figure 3). We also examined, for the first time, the likely interaction between IAV and VCP, which contains CCP modules (4) like human factor H. A/England/2009 (H1N1) and A/Hong Kong/1999 (H3N2) strains were used in this study. A comparable dose-dependent binding by factor H and VCP was evident with H1N1 (Figure 3A) and H3N2 (Figure 3B) subtypes. VSV-G pseudotyped lentivirus was used as a negative control, which did not show any binding with either factor H or VCP. The ability of factor H and VCP to interfere with IAV binding to A549 cells was then assessed via the cell binding assay (Figure 4). An increase of IAV binding in presence of factor H and VCP was seen at the concentration of 10 μg/ml for both H1N1 and H3N2 IAV subtypes, and the binding occurred in a dose and calcium dependent manner (Figure 4), suggesting that factor H and VCP enhance binding of IAV to target cells, which in turn interferes with viral infection in a complement-independent manner. The binding of factor H and VCP was more effective in the case of H1N1 than H3N2. We then set up a far western blot to identify viral particles binding to factor H and VCP (Figure 5). In this assay, virus subtypes (H1N1 and H3N2) separated on SDS-PAGE were transferred to a nitrocellulose membrane and were probed with either 10 μg/ml factor H or VCP. The far western blot also revealed the binding of factor H and VCP to HA (~70 kDa), and M1 (~25 kDa) of both H1N1 and H3N2 IAV subtypes (Figure 5). Furthermore, factor H also bound to NA (~55 kDa) of H3N2 strain more strongly compared to H1N1 strain.

### Modulation of IAV Infectivity by Factor H and VCP in A549 Cells

**Figure 6:**
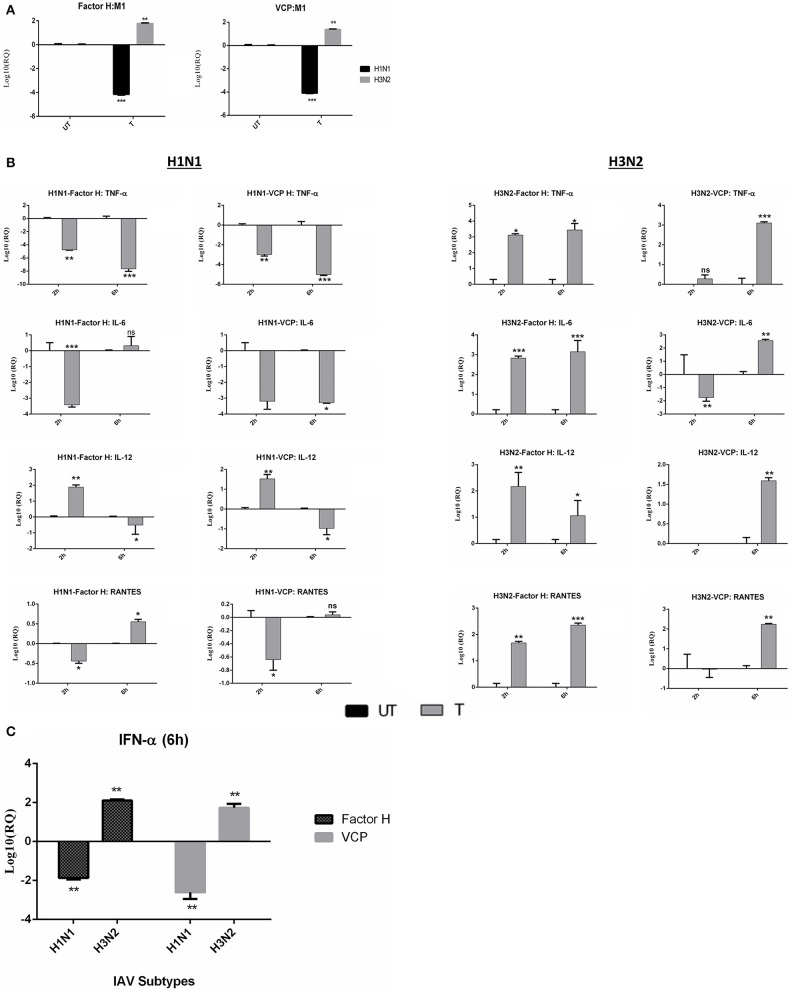
Replication of IAV subtypes and inflammatory responses of A549 cells. **(A)** Following entry inhibition, replication of IAV subtypes (H1N1 and H3N2) is affected by factor H and VCP in human A549 cells. M1 expression was monitored in A549 cells challenged with either H1N1 or H3N2 IAV subtypes (MOI 1) at 6 h. A549 cells were challenged with H1N1 and H3N2 pre-incubated with or without factor H and VCP (40 μg/ml). Cell pellets were harvested at 6 h post-infection. 18S rRNA was used as an endogenous control. Significance was determined using the unpaired one-way ANOVA test (**p < 0.01, and ***p < 0.001) (*n* = 3). **(B)** Transcription expression profile of cytokines and chemokines produced by A549 in response to H1N1 and H3N2 challenge with and without factor H. 18S rRNA expression was used as an endogenous control. Cells only was used as a calibrator sample to calculate relative quantitation (RQ); RQ = 2^−ΔΔCt^. Experiments were conducted in triplicates, and error bars represents ± SEM. Unpaired one-way ANOVA test was used calculate the significance (*p < 0.05, **p < 0.01, and ***p < 0.001) (*n* = 3). (UT, untreated sample; T, treated sample). **(C)** Expression levels of type I interferon alpha (IFN-α) following 6 h treatments with factor H and VCP. 18S rRNA was used as an endogenous control. RQ = 2^−ΔΔ*Ct*^ was used to calculate the RQ value. Significance was determined using the unpaired one-way ANOVA test (**p < 0.01) (*n* = 3).

In view of factor H and VCP binding to key IAV proteins, an infection assay was set up to assess the impact of this interaction on viral infectivity and replication by factor H and VCP. A549 lung epithelial cells, infected with H1N1 and H3N2 with and without factor H or VCP (40 μg/ml), showed differential expression of M1 mRNA levels at 2 and 6 h post-infection (Figure 6). In the case of H1N1, both factor H and VCP led to the down-regulation (−4 log_10_) of viral M1 transcription at 6 h. However, an up-regulation was seen with H3N2 (2log_10_) subtype following factor H and VCP treatment, suggesting that the inhibitory effect by these proteins is strain specific (Figure 6A).

### Factor H and VCP Trigger an Anti-inflammatory Response in the Case of H1N1

qPCR data revealed that factor H and VCP treatment resulted in the modulation of both pro and anti-inflammatory responses (Figures 6B,C). Following factor H treatment of IAV-challenged A549 cells, the mRNA levels of TNF-α were down-regulated at 2 h (−5log_10_) and 6 h (−8log_10_) in H1N1 infected cells (Figure 6B). However, an opposite effect was seen with H3N2 infected cells; the expression level of TNF-α was up-regulated, although not dramatically, at 2 h (3log_10_), as well as at 6 h (3.5log_10_) (Figure 6B). In H1N1 challenged cells, the mRNA levels of IL-6 (−3.5log_10_) and RANTES (−0.5log_10_) were also down-regulated at 2 h time point in the presence of factor H. Conversely, IL-6 (2.5log_10_) and RANTES (1.5log_10_) levels were up-regulated in H3N2 challenged cells at 2 and 6 h (IL-6/3log_10_) (RANTES/2.5log_10_) treatment with factor H. Factor H also down-regulated IL-12 levels in H1N1 (1log_10_) subtype at 6 h, but enhanced in H3N2 challenged cells at both 2 and 6 h. We also show here, for the first time, the effect of VCP, which contains 4 CCP modules like human factor H, on IAV infection; it mirrored the results obtained with factor H (Figure 6B). A greater down-regulation of TNF-α (−5log_10_), IL-6 (−3.5log_10_) and IL-12 (−1.5log_10_) levels were seen at 6 h following VCP treatment when compared to untreated control (cells + H1N1) (Figure 6B). VCP treated H1N1 challenged A549 cells showed a reduced level of RANTES at 2 h (−0.7log_10_), which was raised slightly at 6 h. In the case of H3N2, effects similar to factor H were observed (Figure 6B). TNF-α, IL-6, IL-12, and RANTES were increased at 6 h VCP treatment. Higher levels of pro-inflammatory cytokines, including TNF-α, IL-6, and IFN-α have been detected in IAV infected patients (41), and correlate with severe infectivity. H1N1 infected patients have higher levels of IL-6 in their lungs and serum (42, 43). Therefore, down-regulation of IL-6 by both factor H and VCP in H1N1 infected A549 cells suggests a possible anti-inflammatory role of these two proteins in a strain specific manner. In addition, the ability of factor H (−2log_10_) and VCP (−2.5log_10_) to down-regulate type 1 IFN-α (Figure 6C) at 6 h was also seen in H1N1. However, higher expression levels of IFN-α were detected in the case of H3N2 in the presence of factor H (2 log_10_) and VCP (1.7 log_10_) (Figure 6C).

### Factor H and VCP Act as Entry Inhibitors of H1N1 Viral Infection

**Figure 7:**
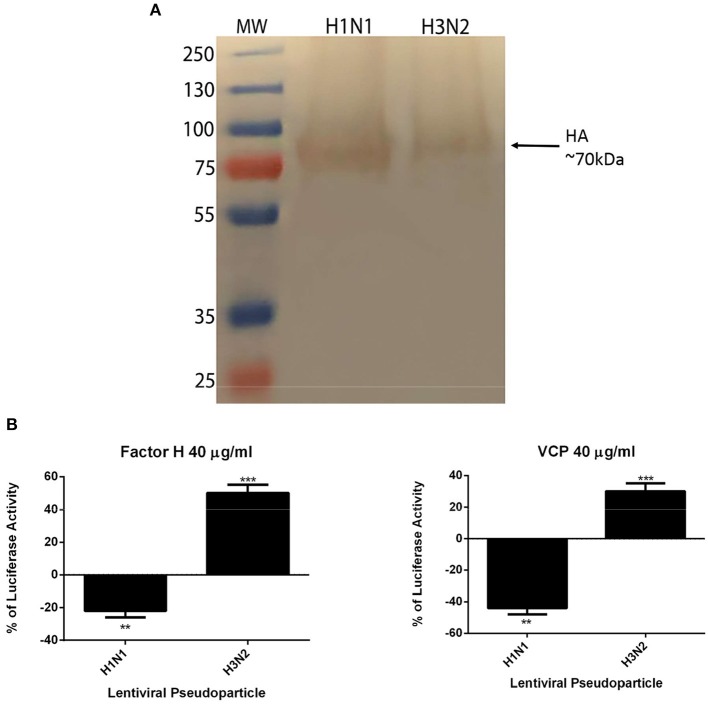
Factor H and VCP inhibit transduction of MDCK cells by H1N1-pseudotyped lentiviral particles. Western blotting to show expression of hemagglutinin (HA) in purified H1N1 and H3N2 pseudotyped lentiviral particles **(A)**. HA expression was evident at ~70 kDa. Luciferase reporter activity of H1N1 and H3N2 pseudotyped lentiviral particles-transduced cells with factor H and VCP **(B)**. Significance was determined using the unpaired one-way ANOVA test (**p < 0.01 and ***p < 0.001) (*n* = 3).

In this study, lentiviral pseudotypes were generated to determine cell entry strategies of H1N1 and H3N2 subtypes of IAV. Production of lentiviral pseudotypes was carried out by co-transfecting HEK293T cells with plasmid containing the coding sequence of IAV glycoprotein combinations such as H1+N1 and H3+N2, pHIV-Luciferase backbone, and psPAX2 plasmids via a calcium phosphate transfection method. Post transfection, H1N1 and H3N2 pseudotyped particles were harvested at 48 h, purified, and analyzed via western blotting. The HA expression level was detected using anti-H1 polyclonal antibody; HA was evident around ~70 kDa (Figure 7A). MDCK cells were transduced with purified H1N1 and H3N2 pseudotyped particles to measure the luciferase reporter activity, with and without factor H or VCP (40 μg/ml). Nearly 25% reduction in the luciferase reporter activity was observed for factor H compared to cells only, challenged with H1N1 pseudotyped particles (Figure 7B). However, factor H enhanced the luciferase activity by 50% in cells transduced with H3N2 pseudotyped particles. In the case of VCP, ~45% reduction of luciferase activity for cells transduced with H1N1-pseudotyped particles was noted, whereas it caused ~30% increase of luciferase activity in cells transduced with H3N2 pseudotyped particles (Figure 7B). Thus, factor H and VCP seem to act as entry inhibitors only in the case of H1N1.

## Discussion

Pathogens have evolved a range of defense mechanisms to overcome the immune response modifying their infectivity and dissemination in the host (44, 45). The complement system, a major arm of the innate immune system, consists of several soluble factors and cell surface receptors that interact with and react to invading pathogens. Some pathogens mimic and recruit complement inhibitors, such as factor H and C4b binding protein (C4BP) to escape complement (46–48).

Factor H is an abundant plasma protein, which has a regulatory function in the homeostasis of the complement system and in the protection of bystander host cells and tissues from injury via the complement activation of the alternative pathway (31). The most important point in the complement activation is the regulation of C3 convertase, which is monitored by factor H, a well-established sialic acid binder. A number of bacteria can recruit factor H via the presentation of specific binding proteins, including factor H binding proteins (fHbp) of *Neisseria meningitidis* (49), CspA and outer surface protein E of *Borrelia burgdorferi* (50, 51), staphylococcal binder of IgG from *Staphylococcus aureus* (52), and pneumococcal surface protein C of *Streptococcus pneumoniae* (53). The factor H ligands, expressed by pathogens, are structurally distinct from each other, but they share the related binding site on factor H that can be localized on a common site of the CCP20 module (45). These interactions increase the binding ability of CCP 19-20 modules to C3b, resulting in a stable complex between factor H, C3b and microbial targets, with an enhanced co-factor activity. *Pseudomonas aeruginosa* express sialic acid on their surfaces, which reduces complement deposition via probable factor H recruitment. Sialic acid also recruits siglec-7/9 leading to immune escape (54). In addition, factor H binds to ApoE on High Density Lipoprotein (HDL) via CCP 5-7 modules, and is used by the bacteria to mimic the plasma HDL to increase their survival in the blood (35).

Here, we investigated the complement-independent function of factor H in the regulation of IAV infection. Since VCP is an efficient complement inhibitor and a homolog of factor H (34), we included it in the present study. VCP is a ~35 kDa secretory protein of vaccinia virus, encoded by the C3L open reading frame (ORF), and contains four CCP modules (34). VCP has been shown to inhibit complement lysis of sheep RBCs, by binding to C3b and C4b (34), and serves as a cofactor in the factor I-mediated cleavage of C3b and C4b (34). Additionally, VCP has been shown to inhibit formation of C3 convertase and accelerate the decay of the alternative pathway (55). In the present study, a direct and complement independent interaction of factor H and VCP with H1N1 and H3N2 IAV subtypes were examined via hemagglutination assay, ELISA, cell binding, and far western blotting. In the hemagglutination assay, factor H inhibited hemagglutination of both H1N1 and H3N2 IAV subtypes; 10 μg/ml being the most effective with H1N1 and H3N2 subtypes.

VCP 4 modules contain a heparin binding site, as does factor H (56). It is possible that in a manner similar to factor H (57, 58), the heparin binding site found within the VCP protein enhances its decay-accelerating action against C3 convertase. SRBCs have high surface sialic acid. Factor H binds to this and protects them from the complement alternative pathway. On removal of the sialic acid with neuraminidase, SRBCs become susceptible to complement lysis. However, other studies suggest that the decay activity rate of VCP does not reduce following neuraminidase-treatment of SRBCs (57, 58), indicating a difference in reaction with sialic acids. Furthermore, a weaker decay rate was seen with CCP 2–4 mutants of VCP. Factor H and VCP seem to bind H1N1 and H3N2 via HA (~70 kDa) and NA (~55 kDa) in addition to recognizing M1. It is well-known that the viral HA can bind to sialic acid residues on surface glycoprotein and its receptor binding depends on the nature of the glycosidic linkage (59). The binding of HA to sialic acids, like factor H, is the initial event in the virus association with human epithelial cells. In addition, the resulting disruption of the neuraminic acid residues can enable the virus to cross over the epithelial cells, thus entering new cells to initiate viral replication.

The immune response of A549 cells following IAV challenge in the presence and absence of factor H or VCP was examined using qRT-PCR. The ability of factor H and VCP to modulate viral replication, due to entry inhibition, was determined using M1 mRNA expression levels between protein treated and un-treated A549 cells challenged with H1N1 or H3N2 subtypes. Both factor H and VCP modulated the IAV replication in a strain dependent manner. In the case of H1N1, both factor H and VCP showed reduced viral M1 expression; however, an increased M1 expression was seen for H3N2, suggesting that the inhibitory effect of these proteins is strain-dependent. This suggests that both factor H and VCP could act as entry inhibitors against H1N1 subtype, but not H3N2.

Several studies have demonstrated both direct and indirect correlation between cytokine levels and viral replication (60, 61). IAV targets lung epithelial cells, and following initial exposure, progeny viral particles proliferate to infect other cells, including alveolar macrophages (62). Thus, an acute inflammatory response is triggered by the activation of pro-inflammatory cytokines and chemokines (62, 63), where higher levels lead to a dramatic cytokine storm; altered levels of TNFs, IFNs, interleukins and chemokines have been detected in IAV infected patients (41). Over-synthesis of IFN levels during early stages of IAV infection causes irreversible lung damage in mice infected with H5N1; however, IFN signaling may also be crucial in preventing H5N1 dissemination (64, 65). TNFs are key soluble factors in the cytokine storm; H5N1 infected mice deficient in TNF receptors, as well as H5N1 infected mice treated with anti-TNF-α antibodies, show no change in survival rate compared to healthy controls (66). Furthermore, IL-6 and IL-1 are the crucial pro-inflammatory cytokines produced by the host during influenza infection. Expression of IL-1 is detected in the early stage of IAV infection, followed by an increased IL-6 expression (67). H5N1 infected mice lacking in IL-6 receptor have demonstrated a poor survival outcome, suggesting the protective role of IL-6 pathway in the cytokine storm (67). Therefore, an unbalanced cytokine storm can also lead to damage in the vascular barrier, causing tissue oedema, capillary leakage, failure of multiple organs, and death (67). However, no specific singular mechanism is reported when it comes to triggering a cytokine storm with respect to influenza strains.

In this study, alterations of cytokine levels were observed in H1N1 and H3N2 challenged A549 cells in the presence of factor H and VCP. Factor H treatment resulted in down-regulation of TNF-α at 2 and 6 h in H1N1 infected cells. In the case of H3N2 infected cells, expression of TNF-α was up-regulated at 2 and 6 h after treatment. Reduced mRNA levels of IL-6 and RANTES were also seen at 2 h treatment with factor H, which slightly increased at 6 h. Conversely, enhanced levels of IL-6 and RANTES were observed in H3N2 infected cells following 2 and 6 h treatment with factor H.

In the case of VCP, TNF-α IL-6, and IL-12 levels were found to be down-regulated at 6 h treatment. H1N1 infected cells treated with VCP also showed reduced levels of RANTES at 2 h. H3N2 infected A549 cells following VCP treatment revealed similar effects as factor H. Up-regulation of TNF-α, IL-6, IL-12, and RANTES were observed at 6 h following VCP treatment. Previous studies have reported enhanced serum levels of pro-inflammatory cytokines, including TNF-α, IL-6, and IFN-α in individuals infected with IAV (68). IL-6 and TNF-α may be the key contributors to virus mediated respiratory diseases, including Acute Respiratory Distress syndrome (ARDS) and acute lung injury (69). During IAV infection, alveolar macrophages are activated, which are the primary phagocytic cells that produce robust amounts of IL-6 and TNF-α (70). Macrophages infected with IAV have also been shown to produce chemokines such as RANTES and monocytes chemotactic protein-1 (MCP-1). This further recruits mononuclear cells to the lungs and facilitates viral clearance (71), and enhances production of those cytokines (e.g., TNF-α, IL-6, RANTES, and IL-8) that are also implicated in the pathogenesis of influenza virus. RANTES, IL-1β, IL-6, and TNF-α induced by influenza result in pro-inflammatory Th1-type immune responses in the infected host (71). Dysregulation of cytokine and chemokine levels during influenza has been demonstrated to promote tissue injury and impaired viral clearance. Additionally, suppression of IFN-α was also evident with factor H and VCP in H1N1 challenged A549 cells. In the case of H3N2, both factor H and VCP increased the expression of IFN-α, which may possibly induce protection of A549 cells against H3N2 viral particles in neighboring as well as non-infected cells. Thus, increased IFN-α by factor H and VCP may also suggest that in the infected A549 cells, factor H and VCP may cause digestion of viral RNA and viral proteins. Down-regulation of IFN-α levels in H1N1 infected cells may suggest the possibility of factor H and VCP treatment to reduce the rate of viral replication. Thus, treatments with factor H and VCP may elicit an anti-viral response, restricting the activation of innate immune cells and associated lung pathology.

Targeting viral entry into a host cell is an emerging approach for developing anti-viral therapy as viral propagation can be either restricted or blocked at an early stage of viral cycle, minimizing drug resistance by released virions. In this study, we have generated lentiviral pseudotypes, a safe surrogate model to mimic the structure and surfaces of IAV in order to determine if factor H and VCP act as an entry inhibitors in cells transduced with pseudotyped IAV particles that are restricted to only one replicative cycle. Factor H treatment resulted in 25% reduction of luciferase reporter activity in MDCK cells transduced with H1N1 pseudotyped particles. However, the addition of factor H increased the luciferase activity by 50% in cells challenged with H3N2 pseudotyped particles. VCP was found to reduce luciferase reporter activity of H1N1 transduced MDCK cells by 45%. As is the case with factor H, VCP also enhanced the reporter activity of MDCK cells transduced with H3N2 pseudotyped lentiviral particles by 30%, suggesting the ability of factor H and VCP to enhance viral infectivity through binding to cell surface bound HA found on infected MDCK cells. Our results are consistent with a previous study showing anti-viral activity of Stachyflin, which was found to be resistant against H3N2 due to differences in potential binding pockets for Stachyflin on HA molecule (72).

In conclusion, data from restriction of M1 mRNA levels, pro-inflammatory cytokine responses, and luciferase reporter activity highlights the potential of factor H and VCP as cell entry inhibitors against the H1N1 IAV subtype. It appears that factor H and VCP restrict viral entry for H1N1, whilst conversely promoting H3N2 entry, which may be due to the inherent antigenic variations in binding sites for HA or NA. Further studies are required to pinpoint the specific molecular interactions of factor H and VCP against HA and NA by investigating a range of IAV strains with antigenic variability. This will not only provide further data on the pathogenesis of IAV infection, but also the utility of factor H and VCP, and their recombinant CCP modules, as possible cell entry inhibitors against IAV.

## Data Availability Statement

The datasets generated for this study are available on request to the corresponding author.

## Author Contributions

VM and PV carried out the key experiments. SS and AT carried out supporting experiments. SA, HK, and KC provided crucial reagents and expertise. RS provided factor H and MRCOX23 hybridoma clones. BN provided viral expertise. FA-M produced VCP. UK led the project. VM and PV prepared the first draft of the manuscript. RS, FA-M, AT, BN, and UK carried out extensive editing and review of the manuscript.

### Conflict of Interest

The authors declare that the research was conducted in the absence of any commercial or financial relationships that could be construed as a potential conflict of interest.
